# D-Serine Production, Degradation, and Transport in ALS: Critical Role of Methodology

**DOI:** 10.1155/2012/625245

**Published:** 2012-09-16

**Authors:** John P. Crow, John C. Marecki, Misty Thompson

**Affiliations:** ^1^Department of Pharmacology and Toxicology, The University of Arkansas for Medical Sciences, 325 Jack Stephens Drive, Little Rock, AR 72205, USA; ^2^J. Thomas May Center for ALS Research and Translational Medicine, The University of Arkansas for Medical Sciences, 325 Jack Stephens Drive, Little Rock, AR 72205, USA; ^3^Department of Molecular Physiology and Biophysics, Vanderbilt University Medical Center, 2215 Garland Avenue, Nashville, TN 37232, USA

## Abstract

In mammalian systems, D-serine is perhaps the most biologically active D-amino acid described to date. D-serine is a coagonist at the NMDA-receptor, and receptor activation is dependent on D-serine binding. Because D-serine binding dramatically increases receptor affinity for glutamate, it can produce excitotoxicity without any change in glutamate *per se*. D-serine is twofold higher in the spinal cords of mSOD1 (G93A) ALS mice, and the deletion of serine racemase (SR), the enzyme that produces D-serine, results in an earlier onset of symptoms, but with a much slower rate of disease progression. Localization studies within the brain suggest that mSOD1 and subsequent glial activation could contribute to the alterations in SR and D-serine seen in ALS. By also degrading both D-serine and L-serine, SR appears to be a prime bidirectional regulator of free serine levels *in vivo*. Therefore, accurate and reproducible measurements of D-serine are critical to understanding its regulation by SR. Several methods for measuring D-serine have been employed, and significant issues related to validation and standardization remain unresolved. Further insights into the intracellular transport and tissue-specific compartmentalization of D-serine within the CNS will aid in the understanding of the role of D-serine in the pathogenesis of ALS.

## 1. Introduction 

Of the D-amino acids known to exist in mammals, D-serine appears to be the most abundant, and certainly the most biologically active. Activation of NMDA receptors (NMDA-Rs) requires binding of both glutamate to the NR2 subunit and its coagonist D-serine to the NR1 subunit [1]. (The co-agonist was initially thought to be glycine, hence the older nomenclature of “glycine_B_” site.) Not only is D-serine binding necessary for receptor activation, but it also increases the affinity of the receptor for glutamate and modulates receptor function by decreasing receptor desensitization while promoting receptor turnover via internalization [2]. Given the widespread distribution of NMDA-Rs in the mammalian CNS, and the abundance of glutamate, the requirement for a co-agonist may represent one of nature's safety mechanisms, designed to prevent overstimulation and excitotoxicity. Thus, it is somewhat ironic that D-serine may actually be a primary cause of excitotoxic neuronal death in ALS. By virtue of its ability to increase glutamate binding affinity, D-serine may produce excitotoxic motor neuron death even in the absence of changes in glutamate levels *per se*.

## 2. Regulation of D-Serine *In Vivo*

Despite notable progress in the 20 years since D-serine was first identified in rat brain [3], the mechanisms of production, degradation, transport, storage, and release of D-serine remain enigmatic. Serine racemase (SR) is considered to be the primary endogenous source of D-serine (using L-serine as a substrate), while D-amino acid oxidase (DAO) is generally regarded as the primary mechanism of degradation. This otherwise straightforward view is dramatically confounded by the fact that SR has an alpha, beta-eliminase activity that is 3-4-fold more efficient (Kcat/Km) than its racemase activity [4]. That is, SR produces D-serine from L-serine, but simultaneously degrades both D-serine and L-serine irreversibly to pyruvate and ammonia. To our knowledge, there is no other mammalian enzyme capable of making a product, while at the same time degrading both its product and substrate. Referring to SR as a “strange” enzyme is an understatement [5]. SR knockout mice were originally created to examine the role of D-serine in diseases like schizophrenia [6], but it may be that schizophrenic behavior is best exemplified by SR itself. 

Examination of the reaction mechanism reveals how and why SR has simultaneous, constitutive racemase and eliminase activities. Both reaction pathways of SR share the same resonance-stabilized carbanion intermediate [7, 8]. Whether the reaction proceeds to racemize L-serine to D-serine, or to deamination/elimination (producing pyruvate and ammonia) appears to hinge on whether or not the –OH of Ser84 donates a proton to the intermediate. While it remains possible that some cofactor or other allosteric modulator may serve to switch SR from one activity to the other, all such factors identified to date enhance both activities roughly equally [8]. Wolosker [9] demonstrated *in vivo* biosynthesis of D-serine via purification of serine racemase from 60 rat brains, which included a painstaking examination of low molecular weight cofactors and activity modulators [9]. This was initially done by adding back fractionated tissue supernatants, followed by identification of specific factors (e.g., pyridoxyl phosphate, Mg^2+^, ATP, etc.). Not until three years later did De Miranda et al. (including Wolosker) describe the eliminase activity of SR [10], leading Wolosker to remark in a recent review that “if the elimination reaction had not been discovered *after *the racemization, SR would have been classified as a serine dehydratase enzyme” [8]. The importance of SR-eliminase activity *in vivo* will be discussed later. 

## 3. Impact of SR Knockout on D-Serine Levels *In Vivo*

At least three different SR knockout mouse strains have been created, each using a different strategy, but all targeting the same 37 kD protein [6, 11–13]. In all cases, levels of D-serine in SR knockout mice are lower compared to wild-type mice, but are not zero; in some cases very significant levels remain, particularly in certain brain regions and tissues [6, 11–13]. In the case of the SR knockout mice first reported by Miya et al. [13], no statistically significant change in D-serine levels was seen in cerebellum or in ten other peripheral organs and tissues [14, 15]. Unfortunately, spinal cord was not examined in this study—the most comprehensive to date. Horio et al. [14] did find D-serine levels in cerebellum and peripheral organs to be roughly 10-fold lower than in other brain regions, suggesting that brain regions and organs with lower constitutive levels of D-serine are largely independent of SR. It is noteworthy that cerebellum and spinal cord levels are very similar in humans, and 10-fold lower than in other regions of the CNS [16]. Thus, it is possible that tissues that have constitutively lower levels of D-serine are less dependent on SR-racemase activity for production, at least in the absence of neurological pathology. To our knowledge, our study represents the first measurement of D-serine in spinal cord of SR knockout mice [17]. In that study, knocking out SR had virtually no effect on D-serine levels in spinal cord.

## 4. SR Knockout and D-Serine Levels in ALS

Mutations to Cu, Zn superoxide dismutase (SOD1) were first reported to be a cause of human ALS in 1993, and the G93A SOD1 mutation of SOD1 was overexpressed in mice two years later, yielding the first mouse model of ALS. All subsequent studies on the role of D-serine in ALS have utilized this well-characterized model. The first measurement of D-serine in spinal cord was that of Sasabe et al. [18], who reported that D-serine was 1.5-fold higher in spinal cords of ALS mice; these results were obtained using a DAO-dependent chemiluminescent assay for D-serine (see Section 12). Using a novel D-serine biosensor (see Section 11), we found that cord levels of D-serine were 2-fold higher in G93A mice than in wild type [17], whereas D-serine in brain of G93A mice were the same as wild type. Partial deletion of SR lowered levels significantly, and complete SR knockout further lowered them, but only to levels normally seen in wild-type mice [17]. Thus, deletion *of SR reversed the pathologic increase in D-serine in G93A mice, but not basal D-serine*. Similarly, knocking out SR in mice that did not express mSOD1 had no effect on cord or brain D-serine levels. In contrast to cord, D-serine was not increased in the brains of G93A mice. Ongoing work is aimed at a better understanding of exactly where the different pools of D-serine originate, and what controls D-serine levels in different organs and tissues. However, the results to date do suggest that spinal cord has an absolute requirement for D-serine, but that levels must be carefully maintained within certain limits, perhaps because cord neurons are exquisitely sensitive to it. Why D-serine is elevated in G93A ALS mice is not clear, but may be a consequence of the generalized glial activation that occurs in these mice (see Section 7 for further discussion).

## 5. Phenotypic Effects of SR and D-Serine in ALS 

Sasabe et al. [18] first showed that primary neurons isolated from G93A mouse spinal cords were more susceptible to NMDA-mediated toxicity, in a D-serine-dependent manner. This result helped establish a potential link between D-serine and excitoxicity in ALS. Perhaps more importantly, Sasabe et al. showed an apparent increase in D-serine in one A4V human ALS patient, and two of three sporadic ALS patient cord samples, suggesting that D-serine may play a role in other, non-mSOD1-mediated ALS [18]. The strong correlation between D-serine and ALS in mice (and humans) warranted further work. Thus, we set out to examine a more direct cause-and-effect relationship by crossing SR knockout mice with G93A mice, as well as treating G93A mice with D-serine, to examine the effects of D-serine on disease dynamics. 

Quite unexpectedly, we found that a 50% reduction in SR enzyme (SR+/− : G93A), which lowered D-serine (mentioned above), resulted in earlier disease onset by 13 days [17]. However, once symptoms of motor neuron disease began, disease progression was actually slowed such that these mice lived approximately 10 days longer than G93A littermates with normal SR expression. When examined in terms of survival interval (time from onset to endstage)—a measure of the rate of disease progression—the SR+/− : G93A mice lived for 60.5 days after onset, relative to 38.8 days for untreated G93A mice. This effect on both onset and progression was even more dramatic in G93A mice with complete SR knockout, these mice showed onset at 61 days of age, and endstage delayed to 146 days of age. In this case (compete SR knockout), the survival interval was 85.8 days, that is, mice progressed very slowly over a much longer period than normal. 

Treatment of G93A mice with D-serine was undertaken with the idea that the opposite effects (from SR knockout) would be seen. Despite these expectations, presymptomatic treatment of G93A mice with D-serine (pre-dissolved in chow) had a qualitatively similar effect as with SR knockout; earlier onset and somewhat slower progression. Treatment with D-serine at normal onset (90 days of age) had a “pure” therapeutic effect; progression to endstage was slowed by 19 days. The paradoxical nature of these results began to make sense only when cord levels of D-serine were examined. As mentioned, untreated G93A mice had a 2-fold higher level of spinal cord D-serine. Partial knockout of SR lowered D-serine to levels only slightly higher than those of wild-type mice, and complete SR knockout decreased D-serine levels even more, comparable to those in wild-type mice. Oddly enough, D-serine treatment, either presymptomatically or at onset, had virtually the same effect on D-serine levels as SR knockout, that is, D-serine in cords decreased to those of wild-type mice. Moreover, this effect was only seen in spinal cord, as brains of D-serine-treated mice showed increases of up to 1.5-fold (Thompson, Marecki, and Crow, unpublished). Thus, D-serine added to chow was crossing the blood brain barrier but was somehow being handled differently in cord than in brain.

## 6. Paradoxical Results in G93A Mice

The question naturally arose: how can D-serine treatment produce the same phenotypic changes and result in the same lowering of spinal cord D-serine as SR knockout? The simplest answer was that D-serine degradation had somehow been induced specifically in spinal cord. However, no significant changes in DAO were seen, which could account for this. Thus, the answer must lie elsewhere. The only other known D-serine handling proteins were the transporters—primarily one called aspartate-serine-cysteine-1 or Asc-1. (It should be noted that transporter nomenclature has yet to be standardized, and “ASC”- and “ASCT”-class transporters exist in the literature under a variety of names.) Alanine-serine-cysteine transporter 1 (Asc-1) is a sodium-independent transporter with a relatively high affinity for D-serine [19, 20]. A sodium-dependent transporter similar to the B-type alanine-serine-cysteine transporter, termed ASCT, has a lower affinity for both L- and D-serine and has been observed in astrocytes [19–21]. Asc-1 was first identified on presynaptic neurons, where it takes up D-serine from the synapse, thereby terminating D-serine neurotransmission [20]. In our study, Asc-1 was found to be upregulated in D-serine-treated G93A mouse spinal cords, but not in their brains [17]. Thus, it seemed likely that D-serine might be localized to different cells/compartments as a result of D-serine treatment. However, while changes in the tissue location of D-serine could alter neurotransmission and potentially excitotoxicity, changes in location *per se* could not account for lower whole cord levels of D-serine, unless that change was also accompanied by degradation. Thus, we inferred that the eliminase activity of SR itself was responsible for degradation of D-serine, and Asc-1 was acting to bring D-serine back into the cells where SR was highest. A recent review by Herman Wolosker, who first purified and characterized SR, offered some additional insight [8]. 

## 7. Revised View of Localization of SR and D-Serine: Implications for ALS and Other Disease Conditions

For several years, immunofluorescence studies, based on colocalization of SR with glial markers, have strongly suggested that SR was predominantly found in astrocytes and microglia [22–24]. However, some studies have shown that neurons in some areas of the brain such as the cerebral cortex and hindbrain glutamatergic neurons also contain their own sources of SR [25, 26]. More recent studies, one of which employed an SR knockout mouse as a control [13], as well as an *in situ* hybridization study [27], suggested that SR is predominantly in neurons, not glia [8]. In any case, it appears that neurons cannot make the substrate L-serine and are dependent on astrocytes for a source of L-serine in order to synthesize D-serine *de novo* [28]. Many of the discrepant results could be reconciled if we hypothesized that* glia normally do not express SR, but the presence of ALS-associated SOD1 mutants induces SR expression as a consequence of pathological activation*. This could account for an important finding by Sasabe et al. wherein transfection of isolated microglia with G93A mSOD1 led to apparent expression of SR [18]. This induction would also account for the increase in SR protein [18] and D-serine seen in ALS mouse spinal cord [17, 18], and possible even the lowering of cord D-serine levels following D-serine treatment [17]. 

The location of SR and D-serine, under both normal and pathological conditions, is important not only in terms of enhanced production and potential excitotoxicity, but also in terms of regulation of D-serine levels via SR-eliminase activity. Wolosker makes a compelling argument that* SR-eliminase will ultimately win out in the concurrent battle to both produce and degrade D-serine *[8]. That is, if D-serine remains in the same compartment as SR—an enzyme that degrades both D-serine and L-serine—then D-serine will ultimately be degraded. If the SR was in neurons, both D-serine and L-serine would ultimately be degraded, requiring resupply of L-serine from glia [28]. Transporters like Asc-1 could act to preserve D-serine levels by physically separating from SR-eliminase in neurons. However, if SR is expressed by activated glia in pathologic conditions such as ALS, then Asc-like transporters could do just the opposite—they could serve to degrade D-serine by placing it in proximity to SR-eliminase in the glia. Other Asc-like transporters are only now being identified and, based on the conflicting literature regarding precisely where SR is normally expressed, it is too early to draw any absolute conclusions regarding the role of D-serine transporters in overall regulation, but clearly they could be key to both termination of normal neurotransmission and to excitotoxicity. In any event, it is almost certain that SR plays a critical role in the overall regulation of D-serine, by acting as both producer and destroyer. 

## 8. D-Amino Acid Oxidase (DAO) 

Because D-amino oxidase (DAO) had been known to exist for many decades, it has come to be viewed as the default mechanism for “detoxifying” D-amino acids. In mammals, DAO is relatively nonspecific, acting upon at least seven D-amino acid substrates [29–31]. However, except for D-serine, the D-amino acids known to exist in mammalian systems do not appear to be particularly toxic [32]. Other lines of evidence argue against DAO being the sole mechanism for regulating D-serine levels: (1) the absence of pathologic phenotypes or compensatory changes in mice lacking DAO [5] and (2) the absence of DAO in forebrain, where D-serine exists at relatively high levels [33, 34]. Indeed, careful characterization of a naturally occurring DAO knockout mouse strain suggests that the primary purpose of DAO is to degrade D-amino acids, whether produced endogenously, obtained from the diet, or from bacterial action in the gut, into keto acids that can then be metabolized as energy sources [35]. That is, DAO may act to obtain useful chemical energy from otherwise “inert” amino acids, as much as to detoxify them. 

## 9. DAO Mutations in ALS 

In 2010, Mitchell et al. identified a mutation (R199W) to DAO and reported a high correlation between this mutated DAO and ALS in one family [36]. Because the mutation was found to abolish DAO activity, the immediate inference was that the mutation would result in increased excitotoxic neuronal death via excessive D-serine accumulation. Transfection of NSC34 cells with mutated DAO did reveal significant toxicity, but the D-serine level in the cells was not measured. Thus, no direct correlation between (presumably) higher D-serine concentrations and cytotoxicity could be made. Moreover, an increase in ubiquitinated protein aggregates was seen in the transfected cells. Thus, while this DAO mutation is consistent with the concept of D-serine-mediated toxicity in ALS, it remains to be seen whether spinal cord D-serine levels are indeed altered, as opposed to mutant DAO merely having an increased propensity to misfold and aggregate. 

## 10. Measurement of D-Serine 

Further elaboration of the roles of D-serine in health and disease, and indeed all biologically relevant D-amino acids, will depend on rigorous evaluation, and possibly modification and standardization, of the various measurements techniques and methodologies. Some of the discrepant results reported thus far may be due to comparisons of different methodologies, which may contain artifacts; this is particularly true when indirect, secondary assays are employed, such as the commonly used DAO-based chemiluminescent assay for D-serine. Because this assay is frequently used to measure SR activity as well, the implications of any artifacts extend beyond simple tissue levels of D-serine *per se*. 

HPLC analysis of D-serine and L-serine, based on chiral derivatization to fluorescent species, is clearly the most sensitive and direct way to identify and quantify these low molecular weight (mass = 87.1) amino acids. Because both serine enantiomers are such small, simple molecules, even chiral HPLC columns cannot readily resolve them. Moreover, they lack any type of chromophore, which would permit UV-visible or fluorescent detection. We have employed an existing derivatization technique involving o-phthalaldehyde and boc-L-cysteine to generate a fluorescent, chiral iso-indole [37] and modified the buffer/gradient system (using sodium acetate and acetonitrile) so that D-serine and L-serine could be resolved by more than two minutes (Thompson et al., unpublished). Via the use of authentic D-serine standards added to tissue homogenates, together with DAO pretreatment to remove D-serine (and other D-amino acids), we were able to measure D-serine in mouse tissues and validate an alternate method based on a novel biosensor [17]. Based on our experiences, we feel strongly that HPLC should remain as the gold standard for such analyses and should be used to validate any and all other methodologies, particularly indirect assays that measure secondary enzyme reaction products like hydrogen peroxide or pyruvate. 

## 11. D-Serine Biosensor 

When acting on D-serine, DAO produces hydroxypyruvate, ammonia, and H_2_O_2_. H_2_O_2_ is a very useful product from the standpoint of biochemical analysis, as it can be measured in many ways, including oxidation by an electrochemical probe to generate an electrical current. By encapsulating DAO obtained from the yeast species *Rhodotorula gracilis*—which is more stable than mammalian DAO and has higher specificity and affinity for D-serine [38]—within the tip of an electrochemical probe, D-serine can be selectively and repeatedly quantified very rapidly. Free D-serine diffuses into the probe, is degraded by yeast DAO to yield H_2_O_2_ which, in turn, is oxidized on the metal probe, producing a current that is proportional to the concentration of D-serine in solution [39]. Because the H_2_O_2_ is produced within the biosensor tip, in close proximity (<100 microns) to the electrochemical probe, issues related to H_2_O_2_ dissipation or degradation are eliminated. Also, the presence of any interfering (diffusible) substances, such as H_2_O_2_ formed from other reactions in the medium, are easily controlled via the use of a comparable biosensor containing albumin rather than DAO. Unlike most DAO-based measurements of D-serine, the biosensor provides a rapid measurement that is *not dependent on complete consumption of *all* D-serine present.* Indeed, the amount of D-serine consumed is negligible, such that repeated measurements of the same sample can be made in a very short time. Because the production of H_2_O_2_ near the electrochemical probe reaches steady-state very rapidly—within a few seconds—the biosensor provides repeated measurements essentially in real time. 

## 12. DAO-Based Chemiluminescence Assay for D-Serine 

The most commonly used method for measuring total D-serine in biological samples or *in vitro* reactions is based on DAO-mediated degradation of all D-serine present to hydroxypyruvate and H_2_O_2_, followed by measurement of total accumulated H_2_O_2_ via horseradish-peroxidase- (HRP-) mediated oxidation of luminol [8, 18, 40]. Unlike compounds such as dihydrofluorescein or dihydrorhodamine, which are oxidized to stable fluorescent species (fluorescein and rhodamine, resp.), luminol oxidation yields a transient chemiluminescence signal, which must be collected and quantified in real time in a luminometer. While it is possible to precisely quantify chemiluminescence, other issues exist with this assay, which render it problematic. Firstly, unless yeast DAO is used, there is little specificity for D-serine, thus all D-amino acids present will be measured. While the contribution of other D-amino acids in mouse of human tissue samples will likely be small, it can vary from sample to sample. 

Secondly, accurate D-serine measurements with the DAO/HRP+luminol chemiluminescent assay *require that the DAO reaction go to completion and consume all the D-serine present*—a process that can take a considerable amount of time given the high Km of most DAO enzymes for D-serine [41, 42]. Unless very careful controls are employed, it is not possible to be sure that the DAO/D-serine reaction has gone to completion and, even if it has, it is equally difficult to know whether or not all the H_2_O_2_ produced during the long incubation remains intact, that is, that it has accumulated stoichiometrically with D-serine consumption, and not reacted with some other component or otherwise been degraded. Trace contaminants of catalase (or other peroxidases), redox active metals, or reductants can totally invalidate the results, just as other, unrelated reactions which can produce H_2_O_2_ during or before the extended incubation time, leading to overestimates of D-serine. Quite often the fact that this is a secondary product-type assay is masked when investigators graph results in terms of “D-serine concentration” (*y*-axis) when, in fact, H_2_O_2_ is the substance being measured. In most cases, D-serine concentration is simply inferred as being stoichiometrically equivalent to H_2_O_2_, without employing the controls needed to demonstrate stoichiometry. With regard to controls, this assay must employ dual standard curves—one for authentic H_2_O_2_ and a second for D-serine (plus added DAO). Only then can the true stoichiometry be assessed. Most accounts in the literature make no mention of such controls. 

## 13. Chemiluminescent Assay as a Measure of SR Activity

The shortcomings of the DAO/HRP+luminol chemiluminescent assay for D-serine are marginally manageable for the purpose of measuring total D-serine content in a deproteinated tissue or cell lysate sample. However, when used to assay SR enzyme activity, additional issues come into play, related to the tedious three-step protocol, contamination of commercial L-serine substrate with D-serine, and competing SR-eliminase activity. By its very nature (three distinct enzymatic steps), the SR/DAO/HRP+luminol chemiluminescent assay does not lend itself well to measuring multiple time points at intervals—something which is essential to establishing a true enzymatic rate, that is, linear increases in D-serine concentration over time. Aliquots from an SR reaction with L-serine must be taken at timed intervals, quenched in some way to stop the SR reaction, then incubated for long intervals with DAO to ensure complete D-serine degradation, followed by the addition of HRP plus luminol to measure accumulated H_2_O_2_. When used to measure SR (racemase) activity, this assay typically involves single endpoints determinations of accumulated H_2_O_2_, without consideration for whether production of H_2_O_2_ was linear, whether extraneous H_2_O_2_ or degradation might be occurring, or whether it has accumulated stoichiometrically with D-serine, or how much H_2_O_2_ might have been present at time zero. Regardless of how SR is assayed, multiple time points are essential to determine linearity, or extent of nonlinearity and why. Also, any assay based solely on D-serine will reflect the *net *production of D-serine by SR, that is, the amount produced by racemase activity minus that consumed by SR eliminase activity [4, 43]. 

## 14. Validation of Chemiluminescent Assay 

The accuracy and reliability of the chemiluminescent assay can only be determined by carrying out simultaneous HPLC analysis on the same reaction aliquots and measure both D-serine production and L-serine consumption. Also, standards curves must be done using authentic H_2_O_2_ so that the true stoichiometry between D-serine and H_2_O_2_, under the specific conditions employed, can be determined. Ideally, assays for pyruvate should also be done on the same aliquots. In this way, not only can potential artifacts in the chemiluminescent assay be uncovered, but the relative contributions of SR-racemase and eliminase activities can be quantified. We have carried out such comparisons, and have found the DAO/HRP+luminol chemiluminescent assay to overestimate racemase activity of SR by 10-fold or higher (Marecki, Thompson, and Crow, manuscript in preparation). This appears to be due to high basal levels (at time zero) of H_2_O_2_ and/or production of H_2_O_2_ from other sources during incubation. Again, if time points are collected and analyzed at intervals, including time zero, both in the presence and absence of DAO, then many of these artifacts can be controlled. However, it is not clear from many literature accounts that such controls are employed. Thus, the reliability of many values in the literature cannot be accurately assessed. 

## 15. Chemiluminecsent Assay to Assess SR Kinetics 

Unfortunately, many of the kinetic characterizations of SR *in vitro*, both with enzyme purified from tissues and recombinant enzyme, have involved the use of this problematic chemiluminescent assay. Moreover, because of practical limitations for doing multiple time points, it has typically been used as a single endpoint type assay. That is, L-serine is added to SR reaction mixtures, reactions are allowed to run for 30–60 minutes, and then quenched, followed sequentially by DAO and its cofactor (FAD), and then HRP+luminol on a single endpoint sample. The following assumptions are then made: (1) that D-serine production by SR-racemase was linear over time period employed (SR-eliminase activity is typically ignored altogether), (2) that D-serine degradation by DAO was complete, (3) that the amount of H_2_O_2_ formed is exactly equal to the D-serine present, (4) that no H_2_O_2_ was present at time zero, and (5) that H_2_O_2_ accumulation was not affected by degradation or augmented by production from other sources. If any of these assumptions are incorrect, then D-serine production rates, and kinetic constants calculated for SR, are erroneous. The problem is that, *when single endpoints are examined, it is impossible to assess the validity of any of these assumptions*. A basic axiom of enzyme kinetics is that product must increase over time and that one must base calculations on the linear region where pseudo-first-order kinetics govern enzyme behavior, so-called “initial rates”. At the very least, the rate of enzyme activity over the time period being measured must be known; one cannot measure something at the end and simply assume that the value at the start was indeed zero or that the change in-between was linear over time. In many cases involving the use of this chemiluminescent assay, investigators report that HPLC was used to validate the results, but chromatograms and controls are typically not shown. Even when chromatograms are shown, it is often unclear whether the entire assay was validated and properly controlled, or whether D-serine was simply measured in a few endpoints samples to demonstrate feasibility. 

## 16. Assay of SR via Pyruvate Production

Mindful of the assay issues described above, some investigators have largely avoided them by measuring SR activity via pyruvate production [18, 44–46]. This is, of course, a measure of SR eliminase activity. When used as a generic measure of “SR activity” in tissues or cells, the use of L-serine as the substrate avoids any interference by DAO, but not SR-racemase activity. If D-serine is used as the substrate, then SR-racemase activity is avoided, but controls for potential interference by DAO must be employed. This is easily done via the use of DAO inhibitors like benzoate [17]. Of course, contaminating DAO would not be an issue when assaying recombinant SR. It should be noted that, although the product of the DAO/D-serine reaction is reported to be hydroxypyruvate, we and others have found that a coupled assay employing LDH and NADH works quite well to measure the “pyruvate” product. Also, derivatizing agents known to react with pyruvate, as well as commercial pyruvate assay kits, all seem to work quite well to measure the DAO/D-serine product. Thus, either hydroxypyruvate has similar reactivities to pyruvate (including acting as substrate for LDH) or perhaps spontaneously dehydrates to give pyruvate. 

## 17. Antibody-Based Assay of “Free” D-Serine 

Many investigators have employed an antibody-based technique to visualize D-serine for cell/tissue localization studies (immunohistochemistry) as well as for semiquantitative comparisons [18, 25]. The scarcity of thorough antibody descriptions in more recent publications prompted questions as to how an antibody could be selective for such a small molecule, as well as how a small, soluble molecule would remain inside cells and tissue that had been permeabilized to allow large antibody molecules to enter. These questions were largely answered when it became apparent that the antibody was raised to a glutaraldehyde adduct of D-serine, not to the free amino acid. Thus, in glutaraldehyde-fixed cells and tissues, the antibody appears to be a useful method for visualizing D-serine, both in theory and in practice. It is less clear whether the antibody would work as well when fixatives other than glutaraldehyde are employed, as the resulting D-serine adduct would not be the same. Also, the use of this antibody to quantify D-serine is subject to the same limitations imposed by any type of fluorescent antibody-based technique. That is, careful use of an antibody-based technique can provide semiquantitative comparative data but cannot be relied upon to provide absolute quantitation or to see small changes. 

The real value of the antibody-based measurement of D-serine lies in its ability to determine cellular/tissue localization and, when used is this manner, can provide valuable information that would be lost with other techniques that rely on tissue homogenization. Indeed, no measurement of D-serine in whole tissue homogenates can detect changes in local concentration which may be highly significant, for example, changes in synaptic D-serine. This means that a 2-fold change in* total* spinal cord D-serine, as seen with G93A mice [17, 18], could translate into a much larger change locally. The same is true for changes in protein expression such as Asc-1 transporter in D-serine-treated mice [17] or changes in SR protein when mutant SOD1 is present [18]. Perhaps the best hope for future measurement of local concentrations of D-serine, possibly even in live animals, lies in further refinements of the D-serine biosensor.

## 18. Summary and Conclusions 

D-serine appears to be an important player in both the onset and propagation of mSOD1-mediated ALS and potentially in sporadic ALS as well. The fact that exogenous D-serine treatment produced similar changes to SR knockout in G93A mice, both in terms of phenotype and in terms of lowering cord D-serine levels, strongly suggests that the effects of SR are mediated via its effect on D-serine levels. It remains to be seen whether this is via a true excitotoxic mechanism, but the results to date are consistent with that premise. Although the mechanism is not known, it is clear that D-serine levels are increased by G93A mSOD1, perhaps via generalized glial activation and resulting induction of SR expression. Given what is now known, D-serine may well prove to be a key link between glial activation and motor neuron death in ALS, that is, it may be the elusive mediator of glial-mediated non-cell-autonomous neuronal death. In any case, the production, storage, release, transport, and degradation of D-serine must all be better understood before its complete role in ALS or other neurodegenerative diseases can be fully elucidated. In that regard, the importance of validation and standardization of methodologies for measuring D-serine and the competing activities of SR cannot be overstated. Until such time, questions as to the characterization of SR will linger. In any case, evidence in SR knockout mice indicates that it is not the only source of D-serine in mammals. Ongoing work in our laboratory, both with SR knockout mice (with and without the G93A mSOD1 transgene) and with recombinant SR *in vitro *is aimed at understanding the role of SR-racemase in producing and SR-eliminase in degrading D-serine, as well as other potential sources of D-serine.

**Added in Proof**

A recent study by Sasabe et al. [47] suggests a significant role for DAO in regulating D-serine levels in G93A mice. Measurements of DAO activity in spinal cords of all six mouse phenotypes in our study (17) showed no statistically significant differences. While this suggested that DAO was not the predominant determinant of D-serine levels in G93A mice, measurements were done only on whole cords at endstage disease. Thus, it is possible that different results could be obtained with select (lumbar) cord regions at different ages. Immunohistochemical data from the 2012 Sasabe et al. study did provide evidence for nonneuronal expression of SR in G93A mice, consistent with the notion that SR is induced by glial activation.
